# A Prognostic 5-lncRNA Expression Signature for Head and Neck Squamous Cell Carcinoma

**DOI:** 10.1038/s41598-018-33642-1

**Published:** 2018-10-15

**Authors:** Guancheng Liu, Jinyang Zheng, Liming Zhuang, Yunxia Lv, Gangcai Zhu, Leiming Pi, Junchen Wang, Changhan Chen, Zhexuan Li, Jiangyi Liu, liangjuan Chen, Gengming Cai, Xin Zhang

**Affiliations:** 1 Department of Otolaryngology Head and Neck Surgery, Xiangya Hospital, Central South University, Changsha, 410008 Hunan China; 2 Department of Otolaryngology Head and Neck Surgery, First Affiliated Hospital of Quanzhou, Fujian Medical University, 248 Dong Street, Quanzhou, 362000 Fujian China; 3 Department of Thyroid Surgery, The Second Affiliated Hospital to Nanchang University, Nanchang, 330006 Jiangxi China; 4 Department of Otolaryngology Head and Neck Surgery, The second Xiangya Hospital, Central South University, Changsha, 410010 Hunan China; 5 Quanzhou Disease Prevention and Control Center, 248 Dong Street, Quanzhou, 362000 Fujian China; 6 Department of Pathology, First Affiliated Hospital of Quanzhou, Fujian Medical University, 248 Dong Street, Quanzhou, 362000 Fujian China

## Abstract

Head and neck squamous cell carcinoma (HNSCC) is a common malignant cancer that accounts for 5–10% of all cancers. This study aimed to identify essential genes associated with the prognosis of HNSCC and construct a powerful prognostic model for the risk assessment of HNSCC. RNAseq expression profile data for the patients with HNSCC were obtained from the TCGA database (GEO). A total of 500 samples with full clinical following-up were randomly divided into a training set and a validation set. The training set was used to screen for differentially expressed lncRNAs. Single-factor survival analysis was performed to obtain lncRNAs that associated with prognosis. A robust likelihood-based survival model was constructed to identify the lncRNAs that are essential for the prognosis of HNSCC. A co-expression network between genes and lncRNAs was also constructed to identify lncRNAs co-expressed with genes to serve as the final signature lncRNAs for prognosis. Finally, the prognostic effect of the signature lncRNAs was tested by multi-factor survival analysis and a scoring model for the prognosis of HNSCC was constructed. Moreover, the results of the validation set and the relative expression levels of the signature lncRNAs in the tumour and the adjacent tissue were consistent with the results of the training set. The 5 lncRNAs were distributed among 3 expression modules. Further KEGG pathway enrichment analysis showed that these 3 co-expressed modules participate in different pathways, and many of these pathways are associated with the development and progression of disease. Therefore, we proposed that the 5 validated lncRNAs can be used to predict the prognosis of HNSCC patients and can be applied in postoperative treatment and follow-up.

## Introduction

Head and neck squamous cell carcinomas (HNSCCs) are the most common cancer of the head and neck region^1^. Of these cancers, pharyngeal squamous cell carcinoma (PSCC), laryngeal squamous cell carcinoma (LSCC), and oral squamous cell carcinoma (OSCC) are the most common ones. These cancers account for approximately 5–10% of all cancers and have an average incidence of approximately 10–15 per 100,000 individuals^2^. Moreover, studies have shown an increasing trend in the incidence of a highly malignant form of these cancers in recent years. Despite the rapid development of medical techniques and the continuous improvement of techniques for early diagnosis of HNSCC, advanced cases still account for approximately 50% of clinical diagnoses. Although, surgical procedures, radiotherapy, and chemotherapy have been greatly improved in the past 20 years, but the 5-year survival rate of HNSCC has not been significantly improved, especially for the advanced patients. Therefore, determination of core hallmarks of early-stage cancer is urgently required to improve patient prognosis.

An increasing number of studies have shown that head and neck cancer is a genetic disease in which many oncogenes and tumour suppressor genes participate in a synergistic process involving many stages and pathways^3^. The mechanisms for the pathogenesis and progression of head and neck cancer have been thoroughly studied at the cell and molecular levels, especially at the gene and long non-coding RNA (lncRNA) levels. These studies searched for genes and lncRNAs associated with head and neck cancer and found that some of these genes played important roles in prognosis, treatment, and prevention^4^. Early detection of these genes and markers has resulted in a new method for investigation of the pathogenic mechanisms of head and neck cancer and to increased accuracy of clinical treatment and prognostic evaluation.

With the rapid development of experimental techniques and computational studies for lncRNA discovery, a large number of lncRNAs have been discovered in various eukaryotic organisms. However, the function of lncRNAs in head and neck squamous cell carcinoma remains unintelligible. In particular, there are no robust lncRNA sets to predict the prognosis of HNSCC. Therefore, in this study, we tried to identify essential lncRNAs associated with HNSCC prognosis and construct a powerful prognostic model for risk assessment of HNSCC.

## Results

### Data source and pre-processing

**Figure 1:**
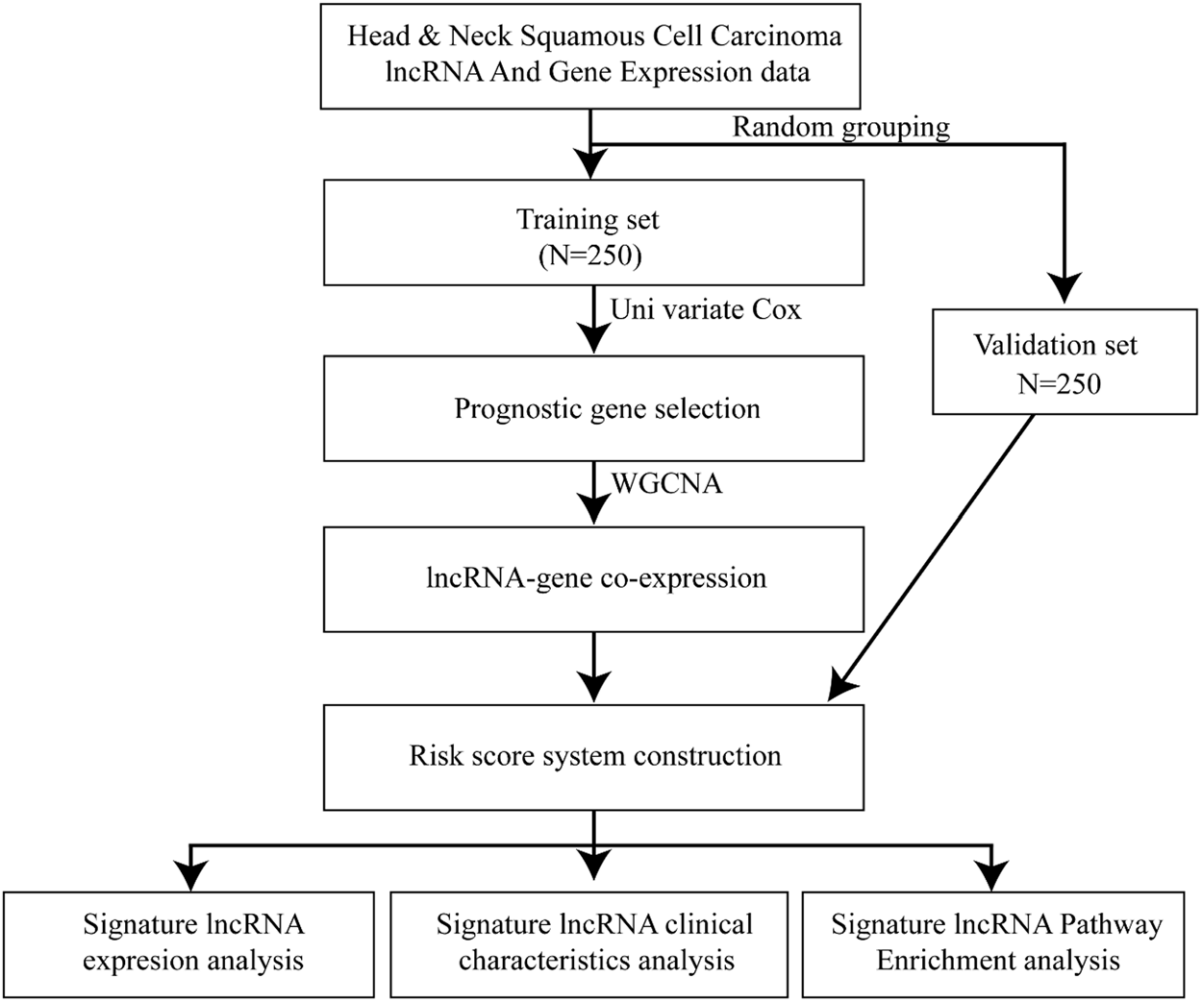
Flowchart of the model construction process. A total of 500 samples with full clinical follow-up were randomly divided into a training set and a validation set. The training set was used to screen for differentially expressed lncRNAs. Single-factor survival analysis was used to obtain lncRNAs associated with prognosis. A robust likelihood-based survival model was constructed to identify lncRNAs that are essential for disease prognosis. A co-expression network of genes and lncRNAs was also constructed to identify lncRNAs co-expressed with genes to serve as the final signature lncRNAs for disease prognosis. Then, the prognostic effects of the signature lncRNAs were tested by multi-factor survival analysis, and a disease prognosis-scoring model was constructed.

A total of 500 head and neck cancer samples and a total of 14448 lncRNA expression values were obtained from TCGA RNAseq data^5^. Then, the 500 samples were randomly and equally divided into a training set and a validation set, as shown in Table 1. The training set was then used to construct the model; Fig. 1 is a flowchart of the model construction process.

**Table 1:** Clinical characteristics of the training set, validation set and entire set.

	Training set (N = 250)	Validation set (N = 250)	Entire set (N = 500)
Age (mean ± SD)	60.99 ± 12.22	61.16 ± 11.62	61.08 ± 11.92
Sex (male/female)	189/61	178/72	367/133
Clinical M (M0/M1)	239/2	231/3	470/5
Clinical N (N0/N1/N2 + 3)	127/36/78	112/44/81	239/81/159
Clinical T (T1/T2/T3/T4)	20/74/59/90	13/69/71/89	33/143/130/179
Clinical stage (I/II/III/IV)	12/49/48/135	7/46/54/135	19/95/102/270
Overall survival time (days)	673 ± 862	662 ± 831	668 ± 846
Status (dead/alive)	76/174	90/160	166/334

### Screening for differentially expressed genes

6654 altered lncRNAs were identified among the 14448 lncRNAs in the training set according to the screening criteria. The expression levels of the 6654 lncRNAs in the 250 samples obtained from screening were subjected to single-factor survival analysis with coxph, and 685 differentially expressed lncRNAs with prognostic significance were identified (p < 0.05, Table 2). The 685 lncRNAs were subsequently used as seed lncRNAs. Table 2 shows the 20 most significant lncRNAs.

**Table 2:** The top 20 lncRNAs with significant effects on prognosis obtained from single-factor survival analysis of lncRNAs with altered expression.

lncRNA	logrank test p value
NCF4-AS1	4.53E-05
RP11-255H23.4	0.00010702
RP11-347C18.5	0.000166483
RP11-197N18.2	0.000279143
RP11-63E9.1	0.00029151
AF064858.6	0.000331408
CTC-499J9.1	0.000452319
RP11-65J21.4	0.000567056
RP11-357H14.17	0.000571739
AC002066.1	0.000588509
AC078883.4	0.000672378
RP11-135A1.3	0.000737051
RP11-121C2.2	0.000755192
LINC00571	0.00076452
RP11-180M15.7	0.000858325
EDRF1-AS1	0.000897423
AC021188.4	0.000998802
LINC01624	0.001009089
LINC00460	0.001140318
AC019048.1	0.001392068

Complete results are shown in Supplementary Table S2.

### Screening for signature lncRNAs that affect prognosis

**Figure 2:**
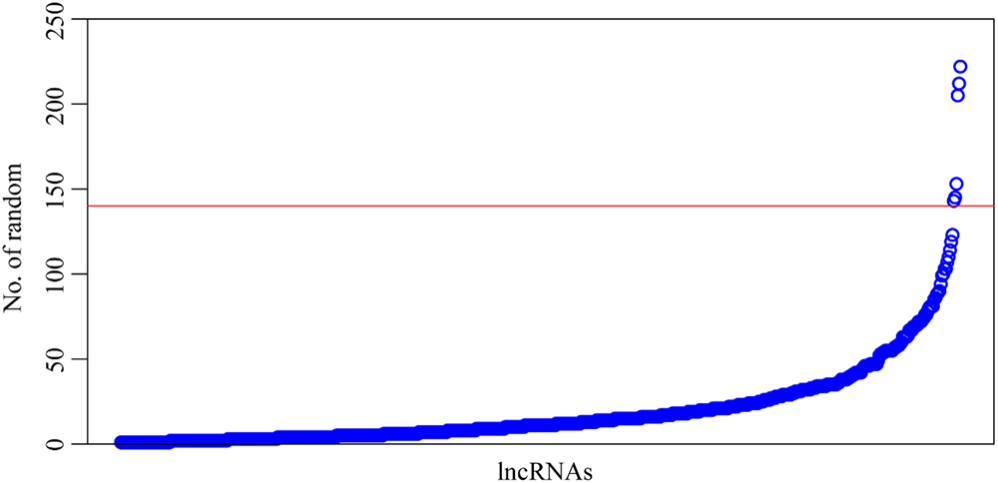
Frequency histogram (1000 cycles) of random lncRNAs. A total of 644 lncRNAs emerged from the results of 1000 cycles of robust likelihood-based survival modelling. Figure 2 shows a frequency histogram of the 644 lncRNAs. The horizontal axis shows all the lncRNAs sorted by frequency from low to high; the vertical axis shows the frequency of the lncRNA in 1000 cycles of robust likelihood-based survival modelling. There was a large gap between lncRNAs with frequencies of 123 and 143. Finally, we selected lncRNAs with a frequency of 143 or more as signature lncRNAs affecting prognosis.

A total of 644 lncRNAs emerged from the results of 1000 cycles of robust likelihood-based survival modelling (Table 3). Table 3 shows the 20 lncRNAs with the highest frequencies. Figure 2 shows the frequency histogram of the 644 lncRNAs. There was a large gap between lncRNAs with frequencies of 123 and 143. Finally, we selected lncRNAs with a frequency of 143 or more as signature lncRNAs that affected prognosis.

**Table 3:** Twenty 20 lncRNAs with the highest frequencies after 1000 cycles.

lncRNA	Count
RP11-347C18.5	222
RP11-474D1.3	212
AC021188.4	205
RP11-197N18.2	153
NCF4-AS1	145
RP11-180M15.7	143
RP11-121C2.2	123
RP11-753H16.3	119
LINC01624	114
RP11-255H23.4	110
EDRF1-AS1	107
AC019048.1	103
RP11-147L13.8	103
RP11-388P9.2	100
RP11-30L15.6	99
RP4-680D5.8	94
RP11-313E19.2	90
RP11-126H7.4	89
RP4-669P10.16	88
SIRPG-AS1	86

### Unsupervised clustering analysis and prognostic signature analysis of the expression profiles of signature lncRNAs

**Figure 3:**
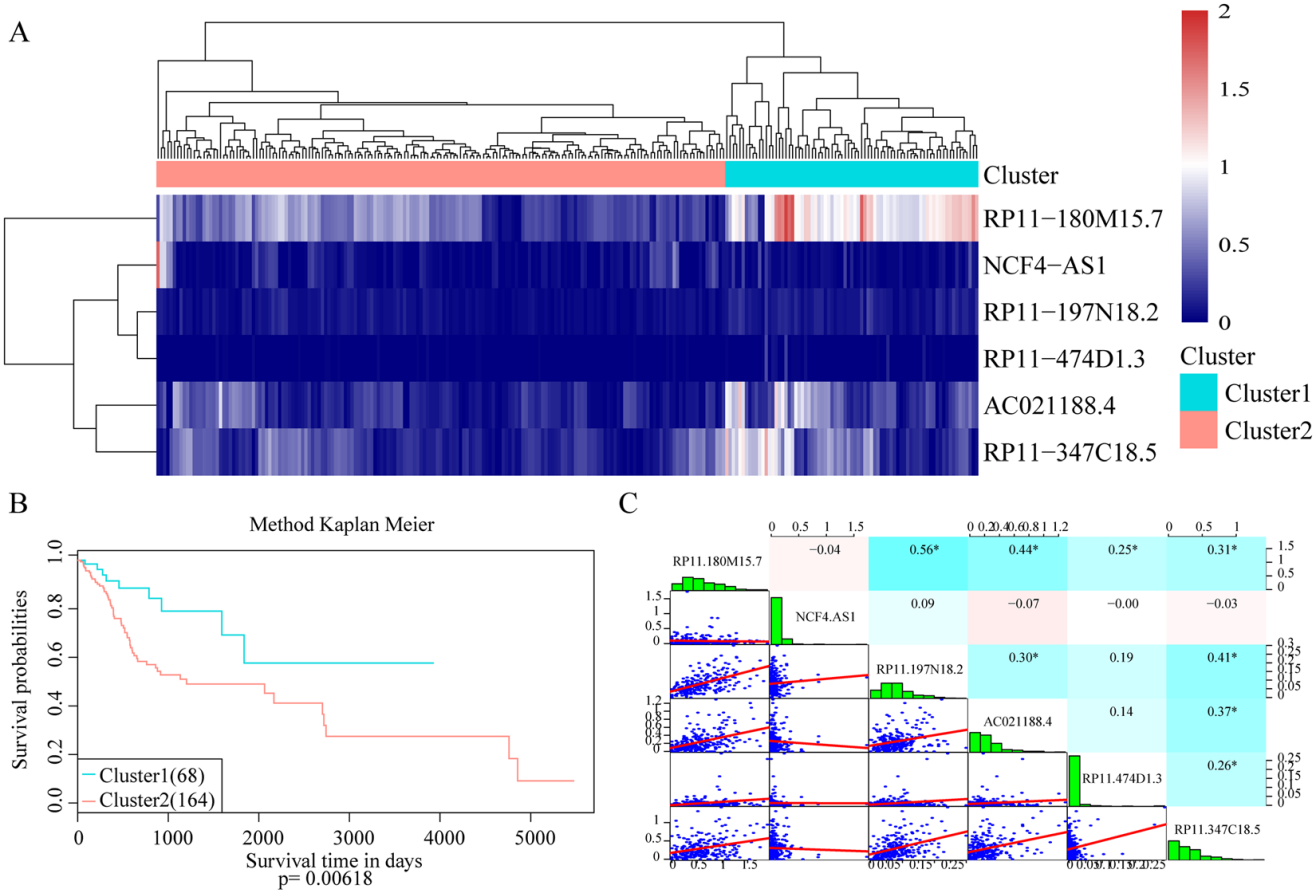
Unsupervised clustering analysis and prognostic signature analysis of the expression profiles of signature lncRNAs. (**A**) Expression profile clustering results of the 6 disease prognostic signature lncRNAs. Values in dendrogram 3A represent the lncRNA expression levels from the hierarchical cluster analysis using Euclidean distances. The horizontal axis represents samples, and the vertical axis represents lncRNAs. Euclidean distance was used to calculate distance. (**B**) Unsupervised clustering yielded the two groups: cluster 1 and cluster 2. The prognostic differences between the two groups was further analysed. (**C**) Correlation analysis of the expression of the 6 lncRNAs. Scatter plots of the expression levels between lncRNAs are presented in the lower left corner. Correlation of expression shown from red to blue with correlation coefficients from −1 to +1 in the upper right corner. A distribution histogram of lncRNA expression is shown along the diagonal (a high-resolution image is presented in Fig. 2).

Six disease prognostic signature lncRNA expression profiles were extracted, and unsupervised hierarchical clustering was performed on the expression profiles of signature lncRNAs. Euclidean distance clustering was used. As shown in Fig. 3A, the expression levels of the 6 lncRNAs were used to divide the samples into two groups, cluster 1 and cluster 2, with 77 and 173 samples, respectively.

Kaplan-Meier survival analysis was used for further analysis of the prognostic differences between cluster 1 and cluster 2 (Fig. 3B). The figure shows that patients in cluster 1 and cluster 2 had significant differences in prognosis, demonstrating that the expression levels of these 6 lncRNAs could be used to effectively distinguish low- and high-risk patients in the clinic. The expression correlation of the 6 lncRNAs was calculated (Fig. 3C). The expression correlation of most of the lncRNAs was low, showing that there was little intersection in the information carried between these lncRNAs, and redundancy was low.

### Construction of the lncRNA-gene co-expression network

**Figure 4:**
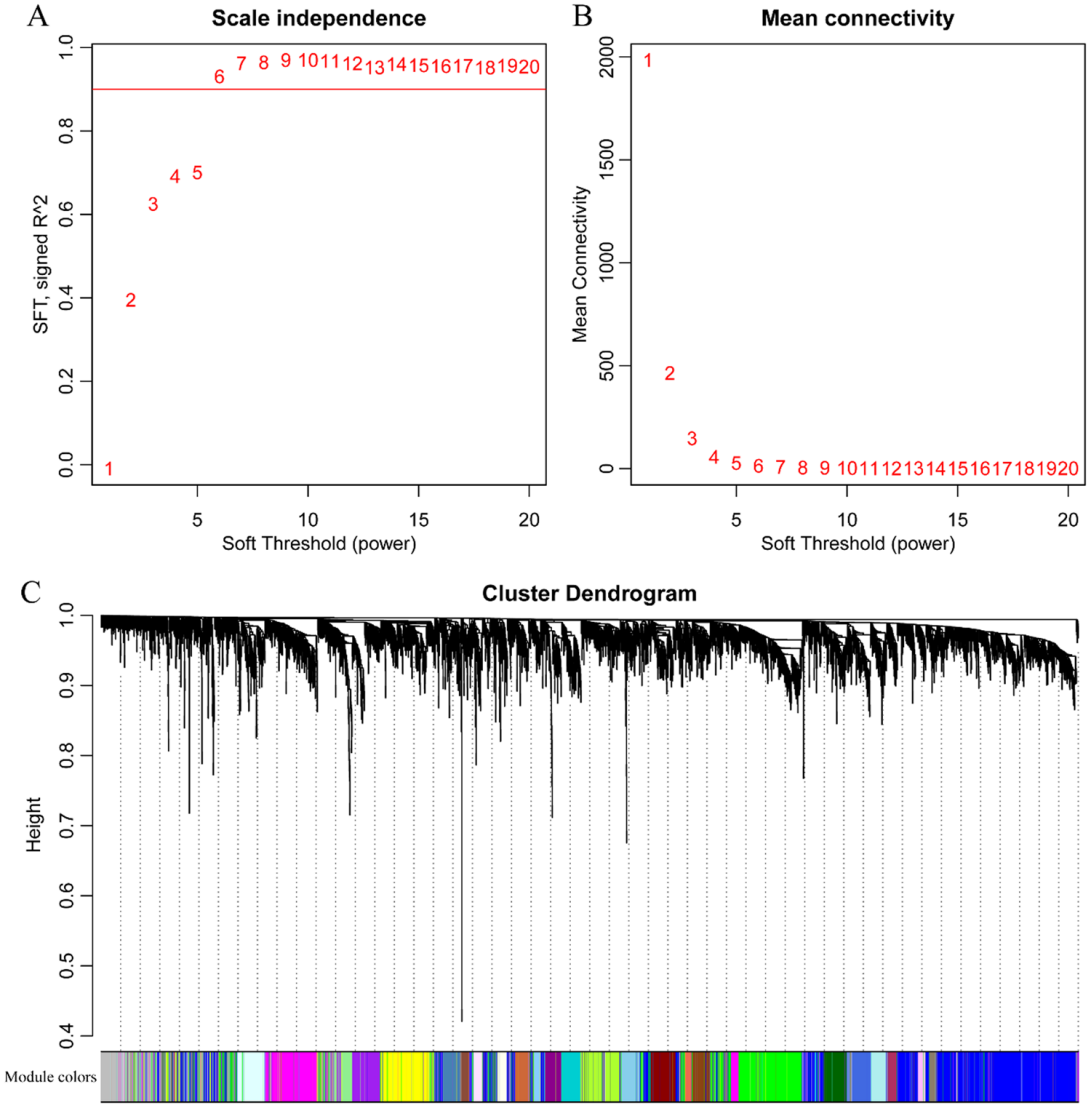
Construction of a lncRNA-gene co-expression network. (**A**,**B**) Depict analyses of network topology for various soft-thresholding powers. (**C**) Depicts a gene dendrogram, and the modules are shown in different colours.

Network construction was performed after combining genes with differential lncRNA expression using the WGCNA R package. Studies have shown that the co-expression network was scale independent, with a correlation coefficient greater than 0.8. We selected the appropriate β value (β = 6) to ensure that the network was scale independent (Fig. 4A,B). Next, the expression matrix was converted into an adjacency matrix, and then the adjacency matrix was converted into a topological matrix. Based on topological overlap measure (TOM), we used the average-linkage hierarchical clustering method to cluster the genes according to the mixed dynamic tree cut standards, and set the minimum number of genes in each gene (lncRNA) network module to 30. After using the dynamic tree cut method to confirm the gene modules, we successively calculated the eigengenes of each module and then performed clustering analysis on the modules. Modules that were close together were combined into new modules, and the height was set to 0.25. A total of 71 modules were obtained (Fig. 4C). Notably, the grey modules could not be clustered with any other modules. Of the 6 lncRNAs, 5 were matched to 3 modules: green (RP11-180M15.7, RP11-474D1.3), magenta (RP11-197N18.2, RP11-347C18.5), and brown (AC021188.4). These 3 modules contained 637, 334, and 752 genes/lncRNAs, respectively.

### Enrichment analysis of the genes in the three co-expressed modules

**Figure 5:**
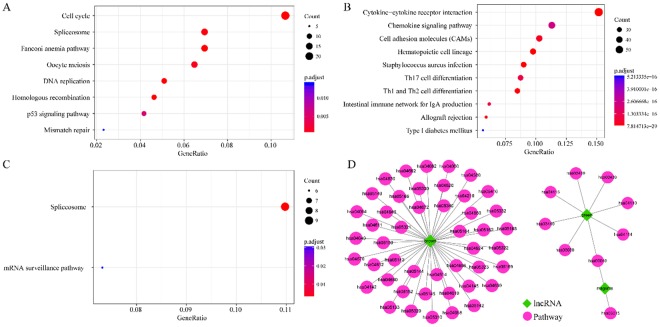
Enrichment analysis of the genes in the three co-expressed modules. (**A**–**C**) Show the most significant enrichment results for the genes in the modules shown in green, brown, and magenta, respectively. (**D**) Shows all enrichment results for the three modules; the lncRNA dendrogram was obtained by average linkage hierarchical clustering. The row of colours underneath the dendrogram shows the module assignment determined by Dynamic Tree Cut.

The clusterProfiler R package was used for enrichment analysis of the genes in the 3 co-expressed modules.Fifty-five KEGG pathways were enriched in the 3 modules, as shown in Fig. 5D, and different pathways were enriched in different modules. There were very few pathways shared between the modules, suggesting that these modules have mutually independent functions. The pathways enriched in the green module were cell cycle, DNA replication, oocyte meiosis, p53 signalling, mismatch repair, and other pathways closely associated with cancer development and progression (Fig. 5A). The pathways enriched in the brown module were associated with signal transduction (Fig. 5B), and those in the magenta module were associated with the spliceosome and mRNA surveillance pathway (Fig. 5C). The pathways enriched in these 3 modules are closely associated with cancer development and progression.

### Prognostic value of lncRNA signatures for assessing clinical outcome of head and neck cancer

A prognostic risk model was constructed from the 5 disease prognostic signature lncRNAs. First, multi-factor survival analysis was used to construct a prognostic risk assessment system from the lncRNAs in the 3 modules using the Equation 1

1 $$\begin{array}{c}Riskscore={}_{-}0.42\ast ExprRP11-180M15.7{}_{-}5.18\ast ExprRP11\\ \,\,\,\,\,-197N18.2{}_{-}1.78\ast ExprAC021188.4{}_{-}30.75\ast ExprRP11\\ \,\,\,\,\,-474D1.3{}_{-}2.64\ast ExprRP11-347C18.5\end{array}$$

**Figure 6:**
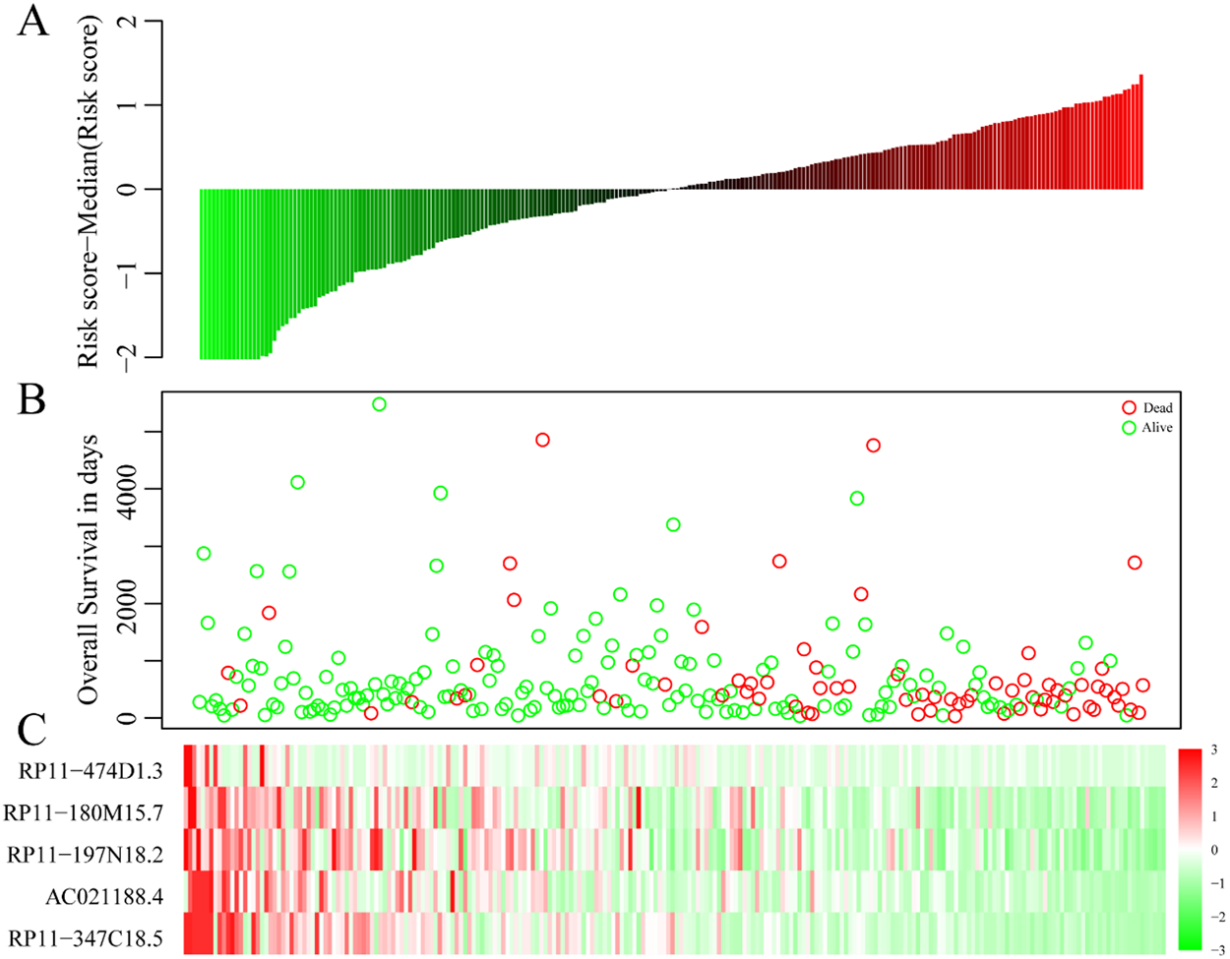
A prognostic risk model was constructed from the 5 disease prognostic signature lncRNAs. The horizontal axis represents samples. (**A**) Samples sorted by risk score; (**B**) Disease prognosis and survival time corresponding to different risk scores in (**A**). Green, alive at follow-up, red, already deceased. The figure shows that as risk scores increased, patient mortality risk increased. (**C**) Expression levels of the 5 signature lncRNAs corresponding to different risk scores in (**A**). The figure shows that as the risk score increased, the expression levels of the 5 lncRNAs gradually decreased.

The concordance index of this model was 0.743, indicating that this model had high reliability. We calculated the risk score for each sample according to the risk assessment model and determined the lncRNA expression status and prognosis associated with different risk scores (Fig. 6). The figure shows that patient mortality risk increased as the risk score increased and that as the risk score increased, the expression levels of the 5 lncRNAs gradually decreased.

#### ROC analysis of the scoring model for screening the best classification threshold values

**Figure 7:**
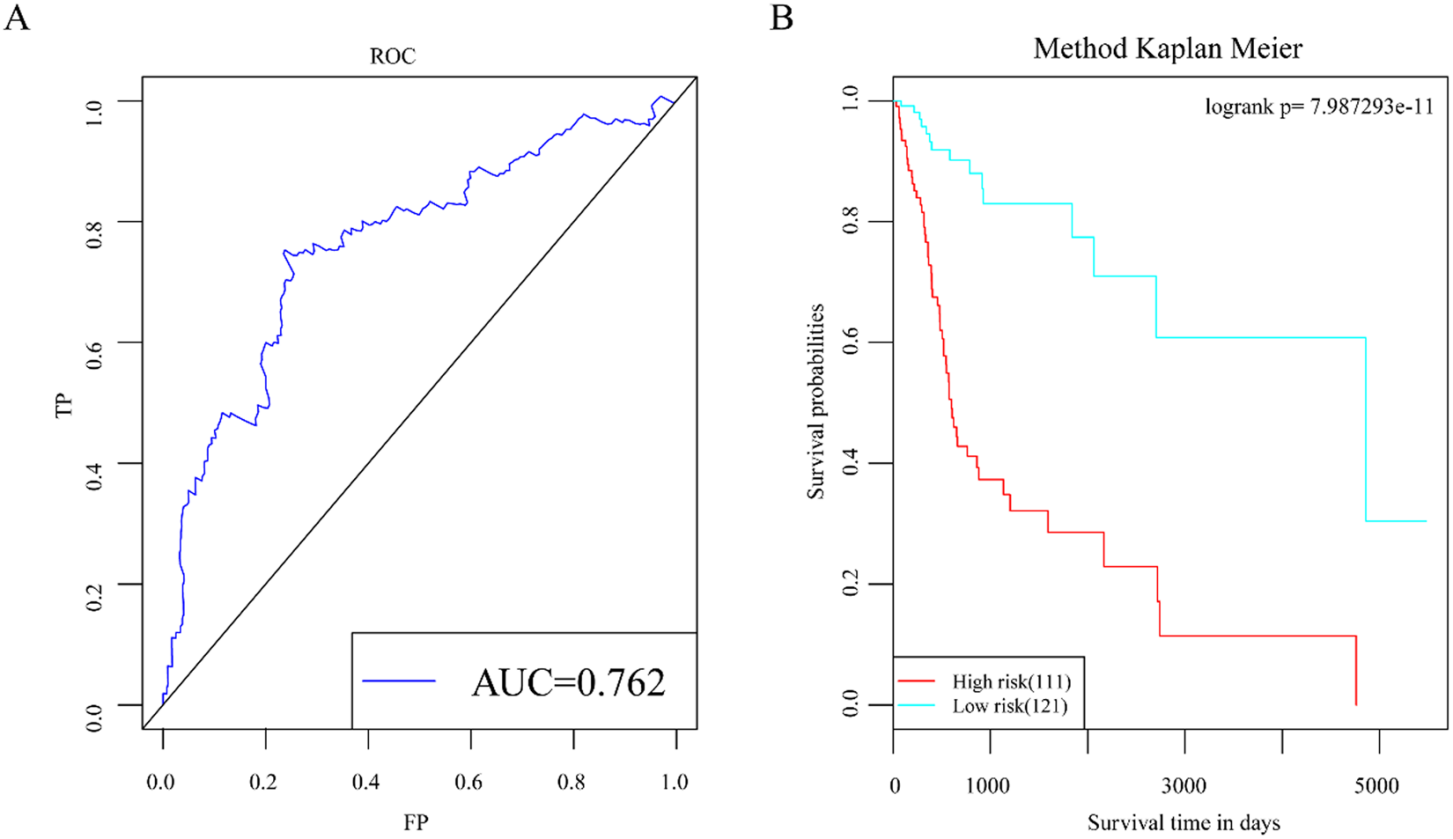
ROC analysis of the scoring model for screening the best classification threshold values. (**A**) ROC curve of the risk score model. (**B**) Prognostic difference analysis after classifying samples into high- and low-risk groups according to the best threshold value.

He risk score of the test set was calculated according to the risk assessment system. The survival ROC R package was used to perform ROC analysis of the risk assessment system^6^. The results in Fig. 7A show that the AUC was 0.762. A best threshold value of -1.47 was further selected for classification, and prognostic difference analysis was performed after classification (Fig. 7B). The results showed that there was a significant difference in prognosis and survival between the high- and low-risk groups.

#### Data validation by the validation set

**Figure 8:**
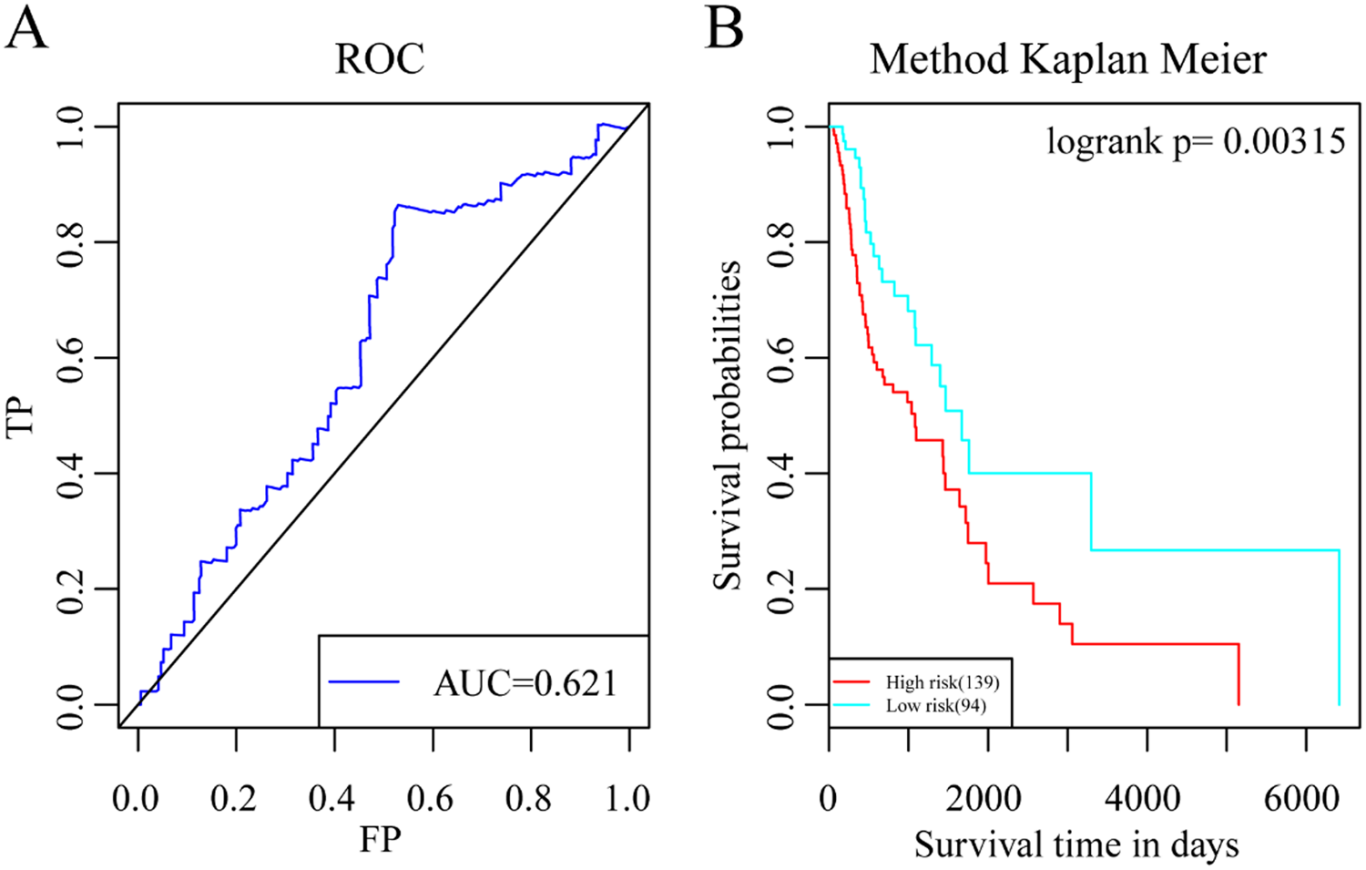
Validation of the 5-lncRNA prognostic model using the validation set. (**A**) AUC curve of the 5-lncRNA prognostic model. (**B**) K-M curve of the prognostic model.

To validate the repeatability and portability of these 5 head and neck cancer prognosis-related lncRNAs, we performed survival analysis using the validation set. Multi-factor survival analysis was performed on the 5 lncRNAs (Fig. 8). The results showed that the 5 lncRNAs also had good classification results with the validation set and that the classification of patient prognosis was highly significant. This finding further showed that the 5 signature lncRNAs screened are essential lncRNAs that significantly affect head and neck cancer prognosis.

#### Expression of the signature lncRNAs in tumour cell lines and tissues

**Figure 9:**
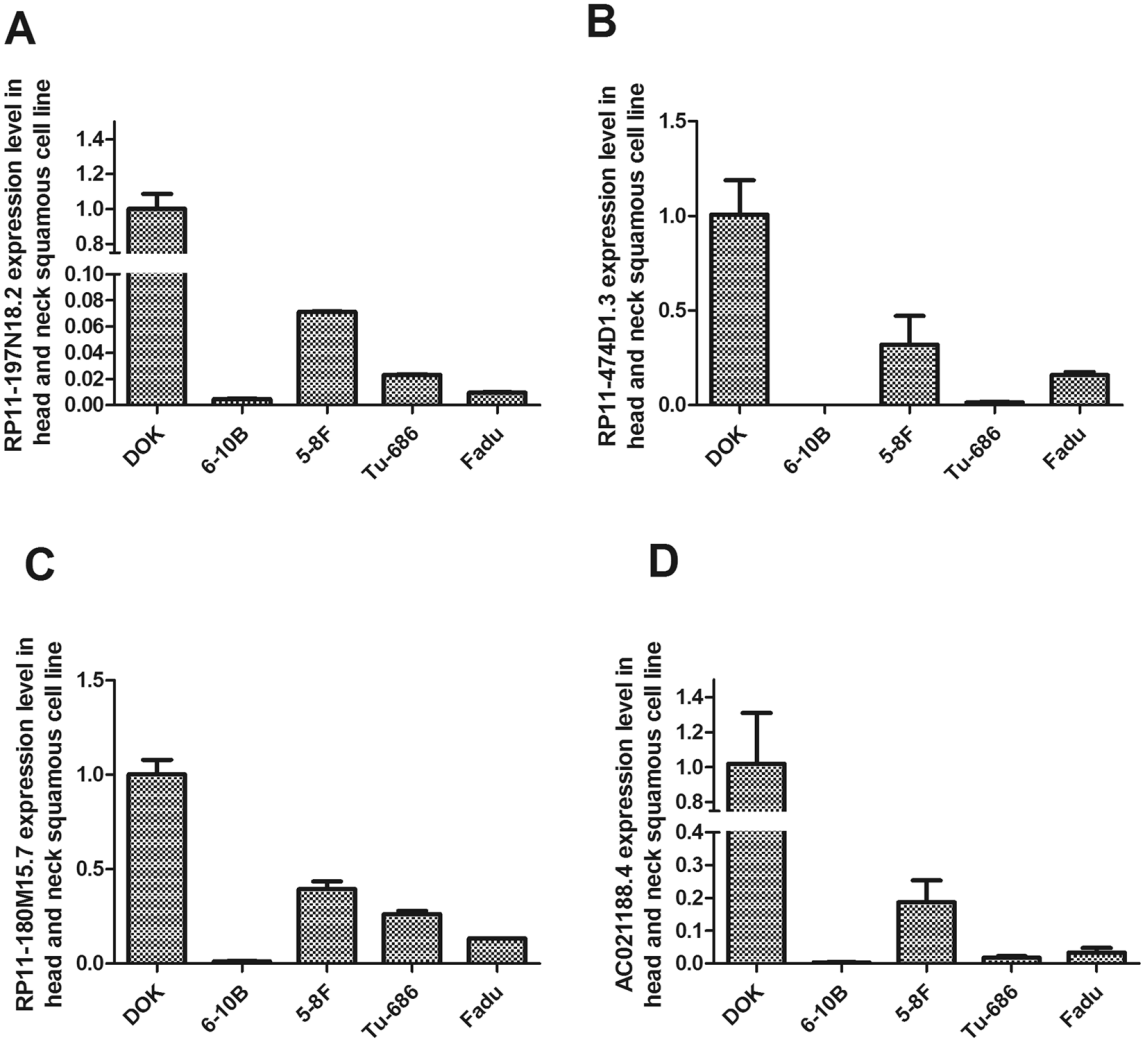
Relative expression levels of four signature lncRNAs in head and neck tumour cell lines. The relative expression levels of four signature lncRNAs in head and neck tumour cell lines (6–10B, 5–8 F, Tu-686 and Fadu) and a human immortalized normal cell line (DOK). (**A**) The expression level of RP11-197N18.2; (**B**) The expression level of RP11-474D1.3; (**C**) The expression level of RP11-180M15.7; (**D**) The expression level of AC021188.4. The results showed that the relative expression levels of the signature lncRNAs were significantly lower in tumour cell lines (6-10B, 5–8 F, Tu-686 and Fadu) than in a human immortalized normal cell line (DOK).

**Figure 10:**
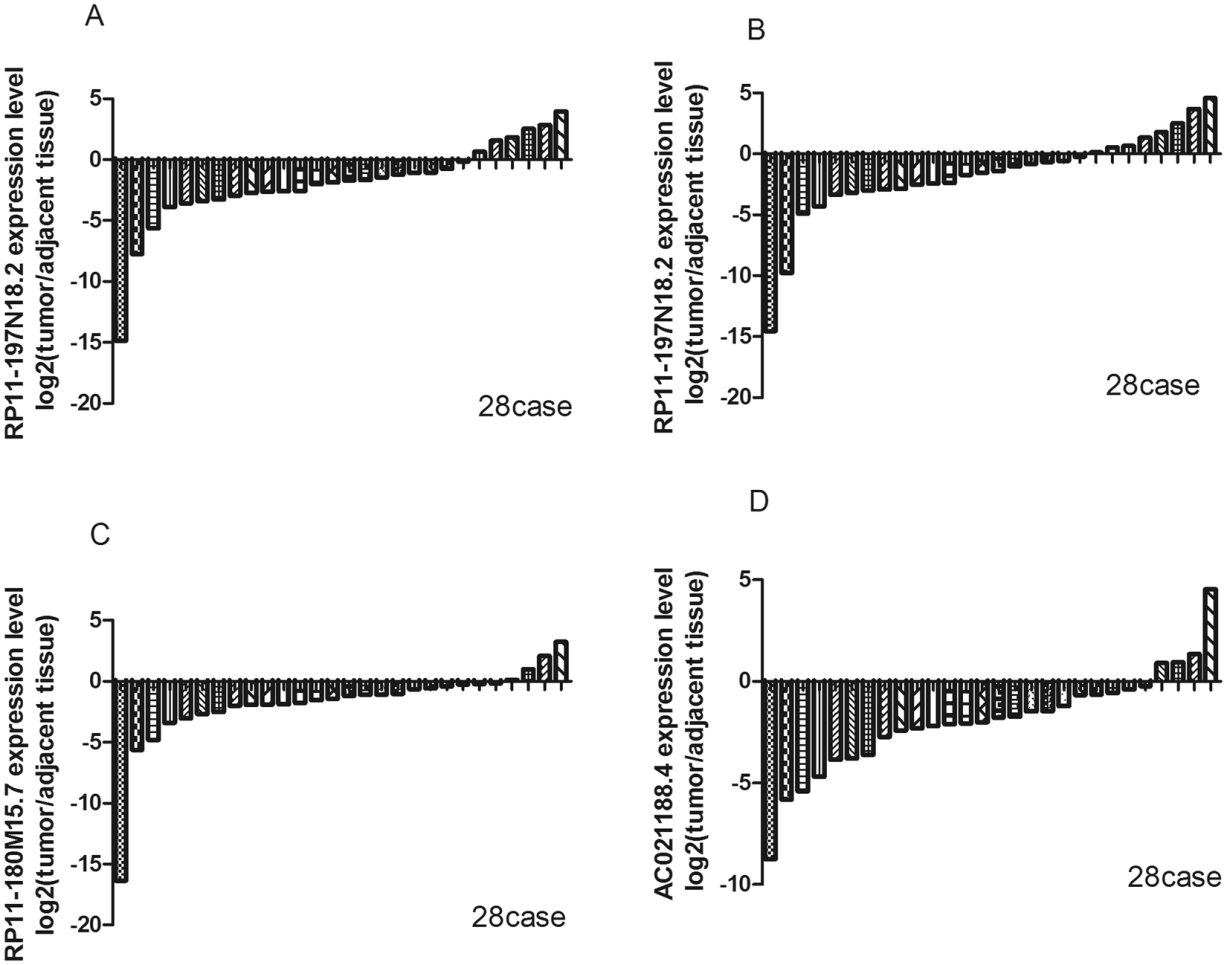
Relative expression levels of four signature lncRNAs in head and neck tumours and adjacent tissues. The relative expression levels of four signature lncRNAs in 28 pairs of head and neck tumours and adjacent tissues. (**A**) The expression level of RP11-197N18.2,it is was down-regulated in tumours in 22 cases; (**B**) The expression level of RP11-474D1.3 was down-regulated in tumours in 21 cases; (**C**) The expression level of RP11-180M15.7 was down-regulated in tumours in 24 cases; (**D**) The expression level of AC021188.4 was down-regulated in tumours in 24 cases. The four signature lncRNAs were significantly down-regulated in tumours compared with the adjacent tissue.

The relative expression level of the signature lncRNAs in tumour cell lines and tissues was verified by qRT-PCR. The results showed that the relative expression levels of the signature lncRNAs were significantly lower in tumour cell lines (6-10B, 5-8F, Tu-686 and Fadu) than in a human immortalized normal cell line (DOK) (Fig. 9). In addition, the four signature lncRNAs were significantly down-regulated in the tumour compared with the adjacent tissue (Fig. 10). We could not determine the relative expression levels of lncRNA RP11-347C18.5 in the tumour cell lines and tissues because no appropriate primers were found for analysis of this lncRNA. Therefore, analysis only four lncRNAs are shown in in Figs 9 and 10.

## Discussion

LncRNAs are defined as RNA molecules greater than 200 nucleotides in length^7^. Due to the special characteristics of lncRNAs, i.e., low expression levels and highly tissue-specific patterns, lncRNAs were previously misidentified as merely “transcriptional noise”. However, accumulating evidence from biological experiments has indicated that lncRNAs carry out various crucial functions, clearly contradicting the conventional viewpoint^8^. An increasing number of studies have shown that lncRNAs are essential factors in the regulation of various cellular processes, including nuclear substructure organization, changes in chromatin state, and regulation of gene expression and activity via interactions with effector proteins^9^. Moreover, recent studies have indicated that lncRNAs play important roles in pathological conditions. Dysfunction of lncRNAs is clearly associated with the development and progression of a wide range of cancers, such as leukaemia, breast cancer, lung cancer, prostate cancer, and ovarian cancer. For example, there is increasing evidence that lncRNAs may exert their effects by regulating protein complexes essential for the regulation of cellular functions and metabolism, and transcription and chromatin state are dynamically regulated by lncRNAs^10–12^. Many reports have already shown that dysregulation of lncRNAs can also affect the regulation of the eukaryotic genome, resulting in cancer progression and uncontrolled growth^13–15^. Therefore, lncRNAs play an important role in cancer and tumour suppressor networks. It has been reported that lncRNAs participate in human cancer progression by regulating cell growth, apoptosis, and invasion^16–18^.

However, the role of lncRNAs in head and neck cancer remains unknown. In particular, there are no robust lncRNA sets to predict the prognosis of head and neck cancer. Fortunately, an increasing number of computational models have been developed to analyse the associations between lncRNAs and disease in recent years. These models provide the most promising lncRNA-disease associations for further experimental validation, hence decreasing the time and cost of biological experiments^19–21^. For example, LRLSLDA is a global ranking approach that can prioritize potential lncRNA-disease associations for all diseases simultaneously. LRLSLDA represents a novel, important and powerful tool in biomedical research for disease treatment and drug discovery, and a cancer hallmark network-based framework for modelling genome sequencing data to predict clonal evolution of cancer and the associated clinical phenotypes was developed by Edwin Wanga *et al*.^22^.

This study screened and analysed for lncRNAs that affect the prognosis of HNSCC using a bioinformatic method, and 5 lncRNAs, namely, RP11-180M15.7, RP11-197N18.2, AC021188.4, RP11-474D1.3, and RP11-347C18.5, were identified. These lncRNAs are closely associated with head and neck cancer prognosis and participate in many KEGG pathways that are involved in cancer development and progression^23^. Moreover, the relative expression levels in the four cancer cell lines, tumours and adjacent tissue are were consistent with previous predictions. There have been very few studies on RP11-180M15.7, RP11-197N18.2, AC021188.4, RP11-474D1.3, and RP11-347C18.5. Zhiqun Li *et al*. found that Homo sapiens 12 BAC RP11-180M15 interacts with the middle hepatitis B virus surface protein using a yeast two-hybrid screen and hypothesized that this interaction was closely associated with the development and progression of different forms of cancer^24^. The other four lncRNAs have not been reported in the literature. Three co-expression modules obtained from enrichment analysis by the clusterProfiler R package showed that pathways closely associated with cancer development and progression were enriched, such as signal transduction, cell cycle, DNA replication, oocyte meiosis, the p53 signalling pathway, mismatch repair, the spliceosome, the mRNA surveillance pathway. We constructed a prognostic risk model using these 5 disease prognostic signature lncRNAs. This model can effectively assess prognostic differences in patients. Simultaneously, the validation set data were used for survival analysis. The results of multi-factor survival analysis of the 5 lncRNAs in the validation set also showed effective classification, which is highly significant for patient prognosis classification. The results of our study show that the 5 lncRNAs are essential lncRNAs that significantly affect head and neck cancer prognosis.

## Materials and Methods

### Data download and pre-processing

Head and neck cancer RNAseq expression profile data were downloaded from the TCGA database. The database contained a total of 500 samples with clinical and follow-up data, from which coding genes and lncRNAs were isolated. Simultaneously, the samples were randomly divided into a training set and a validation set. The training set was used to construct the model, and the validation set data were used as external data to validate the effectiveness of the model^25^.

### Initial screening of differentially expressed lncRNAs in cancerous tissues from head and neck cancer patients

Survival time and lncRNA expression level are closely associated among different patients with the same disease. First, we needed to screen for lncRNAs that strongly interfered with expression in different patients and for lncRNAs that exhibited differential expression in disease samples. The criteria for these lncRNAs was according to the report of Li, J.^25^.

### Seed lncRNA screening

Survival analysis refers to the analysis and inference of animal or human survival time based on data obtained from experiments or surveys and is a method for studying the relationship between many influencing factors and survival time, endpoint, size and extent.

We used the survival R package to perform single-factor survival analysis on the lncRNAs obtained from disease samples that met the criteria for change and selected lncRNAs with a significance level of p < 0.05 as seed lncRNAs^26,27^.

### Screening of key prognostic lncRNAs

There were excess seed lncRNAs obtained from preliminary screening, making it difficult to use these lncRNAs for clinical diagnosis. We constructed a robust likelihood-based survival model to screen signature lncRNAs using the rbsurv R package^28,29^. The procedure was according to the report of Zhiqiang Wang^30^.

We randomly selected 125 samples for 1000 cycles of robust likelihood-based survival modelling. The several lncRNAs with the highest frequencies that emerged were designated the final prognostic signature lncRNAs.

#### Expression profile clustering of prognostic signature lncRNAs

The samples were sorted using unsupervised hierarchical clustering according to the expression profile of the signature lncRNAs. Kaplan-Meier survival analysis was used to further sort prognostic differences among samples^31^.

### Construction of a gene-lncRNA co-expression network

Weighted gene co-expression network analysis (WGCNA) is a systems biological method that uses gene expression data to construct a scale-independent network. The basic concept was as follows^32^: First, a gene expression similarity matrix was constructed by calculating the absolute value of the Pearson correlation coefficients between pairs of genes. The Pearson correlation coefficient between gene i and gene j was calculated using Equation 2, in which i and j represent the expression of the ith and jth genes, respectively.

2 $$Sij=|\tfrac{1+cor({x}_{i}+{y}_{j})}{2}|$$

Next, Equation 3 was used to convert the gene expression similarity matrix into an adjacency matrix. The graph type was signed. In this equation, β is the soft threshold, which is actually the Pearson correlation coefficient of each pair of genes raised to the power of β. This step can strengthen strong correlations and weaken weak correlations from the index scale.

3 $$aij={|\frac{1+cor(xi+yj)}{2}|}^{\beta }$$

Next, Equation 4 was used to convert the adjacency matrix into a topological matrix. TOM was used to describe the degree of association between genes.

4 $$TOM=\frac{{\sum }_{u\ne ij}{a}_{iu}{a}_{uj}+{a}_{ij}}{\min ({\sum }_{u}{a}_{iu}+{\sum }_{u}{a}_{ju})+1-aij}$$

1-TOM represents the degree of dissimilarity between gene i and gene j. 1-TOM was used as the distance for hierarchical clustering of genes. Next, the Dynamic Tree Cut method was used to distinguish between modules. The most representative gene in each module was designated the module eigengene (ME), which represented the overall gene expression level of that module; the ME was the first principal component of each module. Equation 5 was used to calculate the ME, where i represents a gene in module q, and l represents the microarray sample of module q.

5 $$ME=princomp({x}_{ij}^{(q)})$$

We used the Pearson correlation coefficient between the expression profile of a given gene among all samples and the expression profile of the ME to measure the membership of the gene in the module; this is known as module membership (MM). Equation 6 was used to calculate MM, where represents the expression profile of the ith gene, which represents the ME of module q, and represents the membership of gene i in module q. = 0 indicates that gene i is not present in module q, and the closer is to +1 or −1, the more closely gene i is associated with module q. The sign indicate whether gene i is positively or negatively correlated with module q.

6 $$M{M}_{i}^{q}=cor({x}_{i},\,M{E}^{q})$$

Gene significance (GS) was used to measure the degree of association between a gene and external information. Higher values of GS indicate that the gene has greater biological significance. GS = 0 indicates that the gene does not participate in the biological question of interest.

We selected expression data for differentially expressed lncRNAs and differentially expressed genes. The WGCNA R package was used to construct a weighted co-expression network. A soft threshold of 6 was selected for screening of co-expressed modules.

#### Co-expression module enrichment analysis

To determine the functions of lncRNAs involved in each co-expression module, we used the clusterProfiler R package to perform KEGG pathway enrichment analysis on each module^33^.

### Risk assessment model construction and evaluation

Multi-factor Cox regression was used on the obtained prognostic signature lncRNAs participating in co-expressed modules^34,35^. A patient risk assessment system based on the regression coefficients combined with lncRNA expression weighted by the regression coefficients was constructed, and the risk score for each patient was obtained. In other words, the risk score was the linear combination of the lncRNA expression values weighted by the regression coefficients. The risk assessment score of each patient was calculated according to Equation 1. Simultaneously, we used the β value obtained from the training set to assess risk in the cancer patients in the validation set.

### Correlation analysis between the risk assessment model and clinical characteristics

The risk score of each sample was calculated according to the risk assessment system. Using the median risk score as the boundary, the samples were divided into high-risk and low-risk types. In addition, these values were combined with the corresponding clinical characteristics of each sample to analyse the relationship between risk score and each clinical characteristic.

### Patients and tissue preparation

This study was conducted on a total of 28 head and neck tumour samples, which were histopathologically and clinically diagnosed at Xiangya Hospital, Central South University. For the use of these clinical materials for research purposes, prior consent was obtained from all patients, who provided written informed consent, and all research was performed in accordance with relevant guidelines. This study was approved by the Ethics Committee of the Xiangya Hospital of Central South University (ethics committee reference number: 201512549). The patients included 26 males and 2 females. None of the patients had a history of previous malignancies, radiotherapy or chemotherapy. The clinical information for and pathological characteristics of all patients are summarized in Table 4.

**Table 4:** Clinical clinic features of the 28 patients.

		No. of patients	Percentage (%)
Sex	Male	26	92.8
	Female	2	7.2
Age	40–45	20	71.4
	>45	8	28.6
AJCC clinical stage	I-II	12	42.8
	III-IV	16	57.2
T classification	T1-T2	13	86.7
	T3-T4	15	13.3
Lymph node metastasis	N−	11	39.3
	N+	17	60.7
Distant metastasis	M0	28	100
	M1	0	0

### Cell culture

Four head and neck cancer cell lines (6-10B, 5-8F, Tu-686 and Fadu) and one human immortalized normal cell line (DOK) were used in this study, all of which were cultured in complete medium (RPMI-1640) supplemented with 10% foetal bovine serum (Gibco; Thermo Fisher Scientific, Inc., Waltham, MA, USA), streptomycin (100 mg/ml), penicillin (100 U/ml), 25 mM 4-(2-hydroxyethyl)-1-piperazineethanesulphonic acid (HEPES) and 2 mM glutamine. All of the cell lines were maintained as monolayers in a 10-cm plastic dish and cultured in an incubator with a humidified atmosphere containing 5% CO2 at 37 °C.

### Quantitative reverse transcription polymerase chain reaction (RT-qPCR)

TThe relative expression levels of four signature lncRNAs in head and neck tumours and adjacent tissues were determined using RT-qPCR assays. Total RNA was extracted with TRIzol reagent (Invitrogen; Thermo Fisher Scientific Thermo Fisher Scientific, Inc.), and reverse transcription was performed using the All-in-One First Strand Synthesis Kit (GeneCopoeia, Rockville, MD, USA) according to the manufacturer’s protocol. The primer sequences for RP11-197N18.2, RP11-474D1.3, RP11-180M15.7, and AC021188.4 were determined using Primer Premier 5.0 software (Premier Biosoft, Palo Alto, CA, USA), and glyceraldehyde-3-phosphate hydrogenase (GAPDH) was used as a control. The primer sequences for RP11-197N18.2 were as follows: 5′-CCGGGTTCCCATTCTGCTTC-3′ (sense) and 5′-TCTTCCACAATGACAGCCGC-3′ (antisense). The primer sequences for RP11-474D1.3 were as follows: 5′-ACTTGCGCTTCACACTGGAC-3′ (sense) and 5′-GAAATTCTCCTGCGGGGACC-3′ (antisense). The primer sequences for RP11-180M15.7 were as follows: 5′-CCATCGGGTAGGAAGGTCGT-3′ (sense) and 5′-TCGGACTGAGGGAGTACCCTA-3′ (antisense). The primer sequences for RP11-180M15.7 were as follows: 5′-TACAGAAACAGAGTGGAATCTCCG-3′ (sense) and 5′-TTTTATTCCATGATCAGGCTGTGGC-3′ (antisense). The primer sequences for GAPDH were as follows: 5′-ATCAAGAAGGTGGTGAAGCAG-3′ (sense) and 5′-TGGAGGAGTGGGTGTCGC-3′ (antisense). Products were amplified by PCR using the All-in-One qPCR mix (GeneCopoeia, Rockville, MD, USA) and data was obtained and analyzed with a Applied Biosystems ViiA™ 7 Real-Time PCR system. All RT reactions were performed in triplicate, and experimental procedures of qPCR were based on MIQE guidelines. The relative expression levels determined by the 2^−ΔΔct^ method.
